# A Method of Green Citrus Detection in Natural Environments Using a Deep Convolutional Neural Network

**DOI:** 10.3389/fpls.2021.705737

**Published:** 2021-09-07

**Authors:** Zhenhui Zheng, Juntao Xiong, Huan Lin, Yonglin Han, Baoxia Sun, Zhiming Xie, Zhengang Yang, Chenglin Wang

**Affiliations:** ^1^College of Mathematics and Informatics, South China Agricultural University, Guangzhou, China; ^2^School of Electrical Engineering, Guangdong Mechanical and Electrical Polytechnic, Guangzhou, China; ^3^College of Mechanical and Electrical Engineering, Chongqing University of Arts and Sciences, Chongqing, China

**Keywords:** deep learning, green citrus, YOLO v4, agricultural harvesting robotic, object detection

## Abstract

The accurate detection of green citrus in natural environments is a key step in realizing the intelligent harvesting of citrus through robotics. At present, the visual detection algorithms for green citrus in natural environments still have poor accuracy and robustness due to the color similarity between fruits and backgrounds. This study proposed a multi-scale convolutional neural network (CNN) named YOLO BP to detect green citrus in natural environments. Firstly, the backbone network, CSPDarknet53, was trimmed to extract high-quality features and improve the real-time performance of the network. Then, by removing the redundant nodes of the Path Aggregation Network (PANet) and adding additional connections, a bi-directional feature pyramid network (Bi-PANet) was proposed to efficiently fuse the multilayer features. Finally, three groups of green citrus detection experiments were designed to evaluate the network performance. The results showed that the accuracy, recall, mean average precision (mAP), and detection speed of YOLO BP were 86, 91, and 91.55% and 18 frames per second (FPS), respectively, which were 2, 7, and 4.3% and 1 FPS higher than those of YOLO v4. The proposed detection algorithm had strong robustness and high accuracy in the complex orchard environment, which provides technical support for green fruit detection in natural environments.

## Introduction

As one of the most important fruit production countries, China has the largest citrus industry in the world (Liu et al., 2019). Limited by the complexity of environments in the country, citrus picking in China is still dominated by inefficient manual operation. Therefore, the development of intelligent fruit-picking robots is of great value and significance for alleviating labor shortage, saving labor costs, and promoting intelligent agricultural production. Thus, machine vision is the key technology for the development of the ability of picking robots to achieve precise operation, but the detection accuracy and efficiency of existing fruit-picking robots still need to be improved (Liang et al., 2020).

In the complex outdoor environment, the accurate and effective detection of citrus fruits is the prerequisite for robots to complete picking tasks efficiently. The detection accuracy determines the positioning accuracy, which is of great significance for robots to achieve successful picking. Compared with mature fruits with colors different from the background, green fruits are more difficult to recognize in natural environments. The difficulties are as follows: (1) the natural environment has complex illumination, where different light intensities and uneven illumination at different daytime periods would reduce the quality of image acquisition; (2) citrus fruits have diverse growth states as, under natural conditions, citrus fruits are randomly distributed and overlapped with each other and can thus be easily blocked by background objects such as leaves or branches; (3) in addition to having a similar color to leaves, green citrus fruits occupy few pixels in the image due to their small size in reality, which is difficult to detect. These are the common phenomena in the actual orchard environment, and all of them cause great difficulties for algorithms when accurately detecting green citrus fruits.

In recent years, many scholars have tried to detect green citrus and made some progress in the endeavor. For example, Kurtulmus et al. (2011) proposed the concept of an “intrinsic fruit” and detected green citrus by combining color with circular Gabor texture features, subsequently reaching an algorithm accuracy of 75.3%. Sengupta and Lee (2014) proposed an algorithm using color images to detect green citrus fruits; specifically, combining shape features with Hough circle detection to realize the preliminary detection of potential fruits and used texture features. They also used Hough line detection and Canny edge detection to further remove false positives, resulting in an algorithm accuracy of 80.4%. Zhao and Lee (2016) put forward a green citrus detection method based on the sum of absolute transformation difference (SATD) and used texture features to filter out false positives, achieving an accuracy up to 83.4%. Lu and Hu (2017) proposed a hierarchical contour analysis (HCA) algorithm based on the light distribution on the surface of a fruit to realize the detection of green citrus fruits on trees, with an accuracy of 83.5%, a recall rate of 81.2%, and an execution time of 3.7 s. Lu et al. (2018) proposed a method using texture and intensity distribution to detect green citrus fruits in tree images, with an accuracy of 80.4%. Wang et al. (2018) developed a green citrus recognition and counting method based on the local binary pattern (LBP) features and the AdaBoost classifier, with an accuracy of 85.6%. He et al. (2020) proposed a deep bounding box regression forest (DBBRF) green citrus detection method based on a deep border regression forest. The average recognition accuracy and time were 87.6% and 0.759 s, respectively. In summary, the studies discussed previously mainly combined the traditional image processing methods and the basic characteristics of color, texture, and shape to realize green citrus detection. However, in the complex environment of the actual orchard, this kind of method is prone to inference from other factors, such as natural light, leaf occlusion, fruit overlap, and so on.

In addition, some scholars also used heat map, multispectral, or hyperspectral images to detect green citrus fruits. Okamoto and Lee (2009) achieved the detection of green citrus fruits by combining the hyperspectral camera in the range of 369–1,042 nm and using image processing methods such as noise reduction filtering, labeling, and region threshold segmentation. The detection accuracy of the algorithm for complete fruits was between 80 and 89%. Torres et al. (2019) proposed a green citrus detection method based on two hyperspectral reflectance imaging systems, resulting in a detection accuracy of 96.97%. Although multispectral images and hyperspectral images can provide many clues to green citrus, at the same time, there is still much information redundancy, which may lead to the poor real-time performance of the algorithm. Furthermore, compared with the color image acquisition equipment, a multispectral or hyperspectral camera is generally more expensive. Gan et al. (2018) applied the multimodal imaging method to the visual detection of green citrus for the first time, with green citrus fruits being effectively identified through the fusion of color information with thermal information, resulting in an algorithm recognition accuracy of 95.5%. Despite the high accuracy of this method, its thermal images can be easily affected by other factors such as relative humidity, ambient temperature, and wind, so it is rarely used in actual fruit detection tasks.

With the rapid development of machine learning, some researchers have begun to apply deep learning to the visual inspection of fruits (Kamilaris and Prenafeta-Boldu, 2018). Peng et al. (2018) proposed an improved single shot multi-box detector (SSD) deep learning fruit detection model by replacing the VGG16 backbone network with the ResNet-101 model. In the proposed model, the average detection accuracies of imperial orange and navel orange were 91.57 and 90.69%, respectively. Lv et al. (2019) proposed a citrus recognition method based on the improved YOLOv3-LITE lightweight neural network. The method introduced the generalized Intersection over Union (GIoU) border regression loss function and used the MobileNet-v2 as the backbone network, with an average accuracy of 91.13%. Xiong et al. (2020) proposed a citrus detection network based on Des-YOLO v3, which effectively improved the problem of the missed detection of small targets, and the average detection accuracy of the algorithm was 90.75%. Kang and Chen (2020) proposed an apple detection model named LedNet and an automatic labeling tool. The recall and accuracy of the model were 82.1 and 85.3%, respectively. Yang et al. (2020) proposed an integrated system for citrus and branch detection and size estimation and used the Mask RCNN as the citrus fruit recognition model, with the average recognition accuracy of 88.15%. In summary, the mentioned researches mainly focused on mature fruits; however, green fruits are more difficult to detect because of the color similarity between these fruits and their leaves. Combining color with thermal information, Gan et al. (2018) adopted the Faster RCNN algorithm to identify green citrus fruits, with a recognition accuracy of 95.5%. Xiong et al. (2018) also used Faster RCNN to identify green citrus in natural environments, with an average accuracy of 85.49% on the test set. In Afonso et al. (2020), the Mask RCNN algorithm was used for the detection of tomatoes in images taken in a greenhouse, which detected objects and the pixels corresponding to each object. The above researches showed that, due to the strong feature extraction ability of deep convolution neural networks, fruit recognition studies have gradually shifted their focus to recognition methods based on deep learning.

In short, although many researchers have done extensive work on the detection of green citrus, the accuracy and robustness of most detection algorithms still need to be further improved due to the difficulties of green citrus detection already discussed. Compared with traditional machine learning methods, deep learning algorithms have greater advantages in terms of accuracy and real-time performance. However, few studies have been conducted on the use of deep learning methods to detect green citrus fruits in natural environments. At the same time, the resources that fruit-picking robots can use in the orchard are limited, so it is necessary to improve detection algorithms according to actual needs.

Therefore, based on the deep learning method, this study proposed a visual detection method of green citrus fruits in the natural environment, which can provide technical support for the intelligent picking of other green fruits. The main research work is presented as follows. (1) The green citrus images captured in the complex environment of citrus orchard were collected, classified, and enhanced. (2) Referring to the idea of a weighted bi-directional feature pyramid network (BiFPN), a green citrus detection network, YOLO BP, was proposed to improve algorithm accuracy and real-time performance based on the improvement and compression of the YOLO v4 detection algorithm. (3) The YOLO BP network was trained and three groups of comparative experiments were designed to evaluate the performance of the proposed method.

## Materials

### Image Acquisition

**Figure 1:**
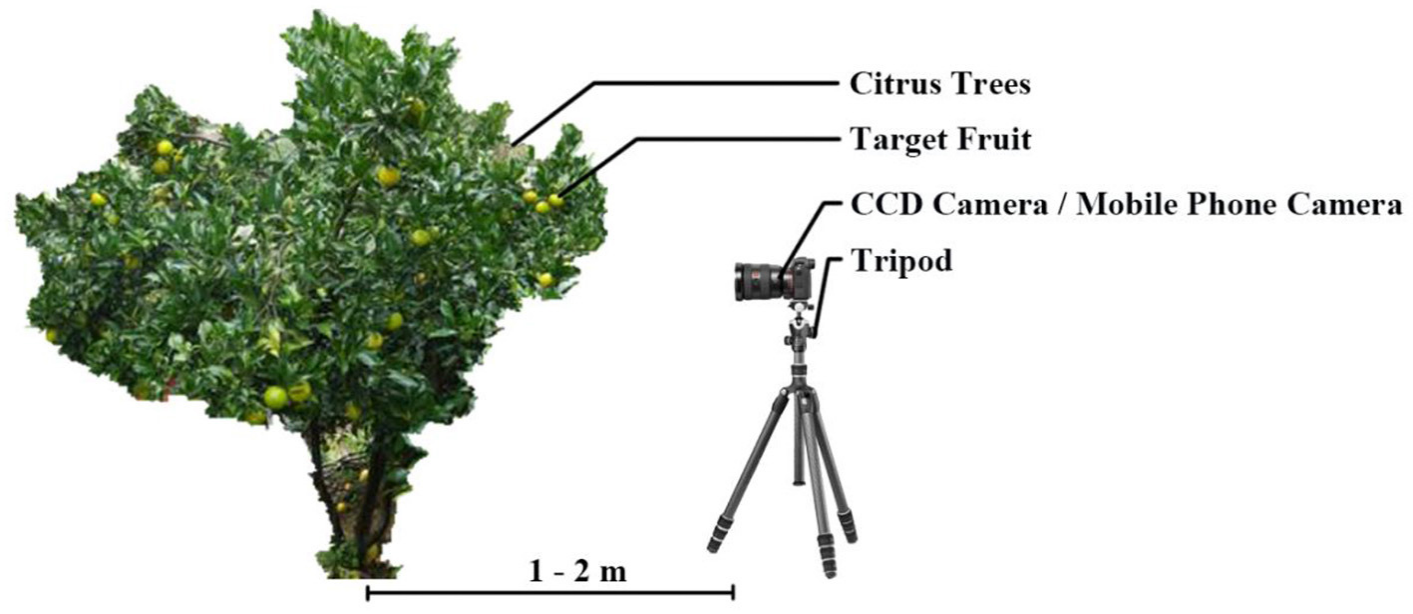
Visual system and image acquisition schematic diagram.

In this study, citrus images were acquired from the citrus orchard of Zengcheng, Guangdong, China. The citrus varieties were emperor citrus and tangerine citrus. The image acquisition experiments were carried out on November 11, 2016, January 8, 2017, and July 20, 2018. As shown as Figure 1, the shooting distance between camera and citrus trunk was between 1 and 2 m during image acquisition, and the citrus fruits are photographed from multiple angles. To meet the diversities of the dataset, a total of 890 green citrus images were taken at different time periods (9:30–17:00) and different light conditions (directional light and backlight) and saved in 24-bit color JPEG format.

The image acquisition system mainly included three cameras, namely, Nikon (Tokyo, Japan), SONY (Tokyo, Japan), and Apple (Cupertino, CA, USA), with the specific parameters of each camera and the corresponding image number shown in Table 1.

**Table 1:** Statistics of the camera parameters and image quantities.

**Camera manufacturer**	**NIKON**	**SONY**	**Apple**
Camera	NIKON D5300	DSC-WX100	Iphone 6s Plus
Pixel resolution	4,496 × 3,000	2,592 × 1,944	3,024 × 4,032
Aperture value	f/5.6	f/5.6	f/2.2
ISO speed	250	160	25
Number of images	278	392	220

### Class Definition for the Green Citrus Scene

As described in the Introduction, except for the color similarity between green citrus fruits and their backgrounds, there are many complex situations in the natural environment, such as light changes, shades of the branches and leaves, overlapping of fruits, changes in the number and size of fruits, etc.

**Figure 2:**
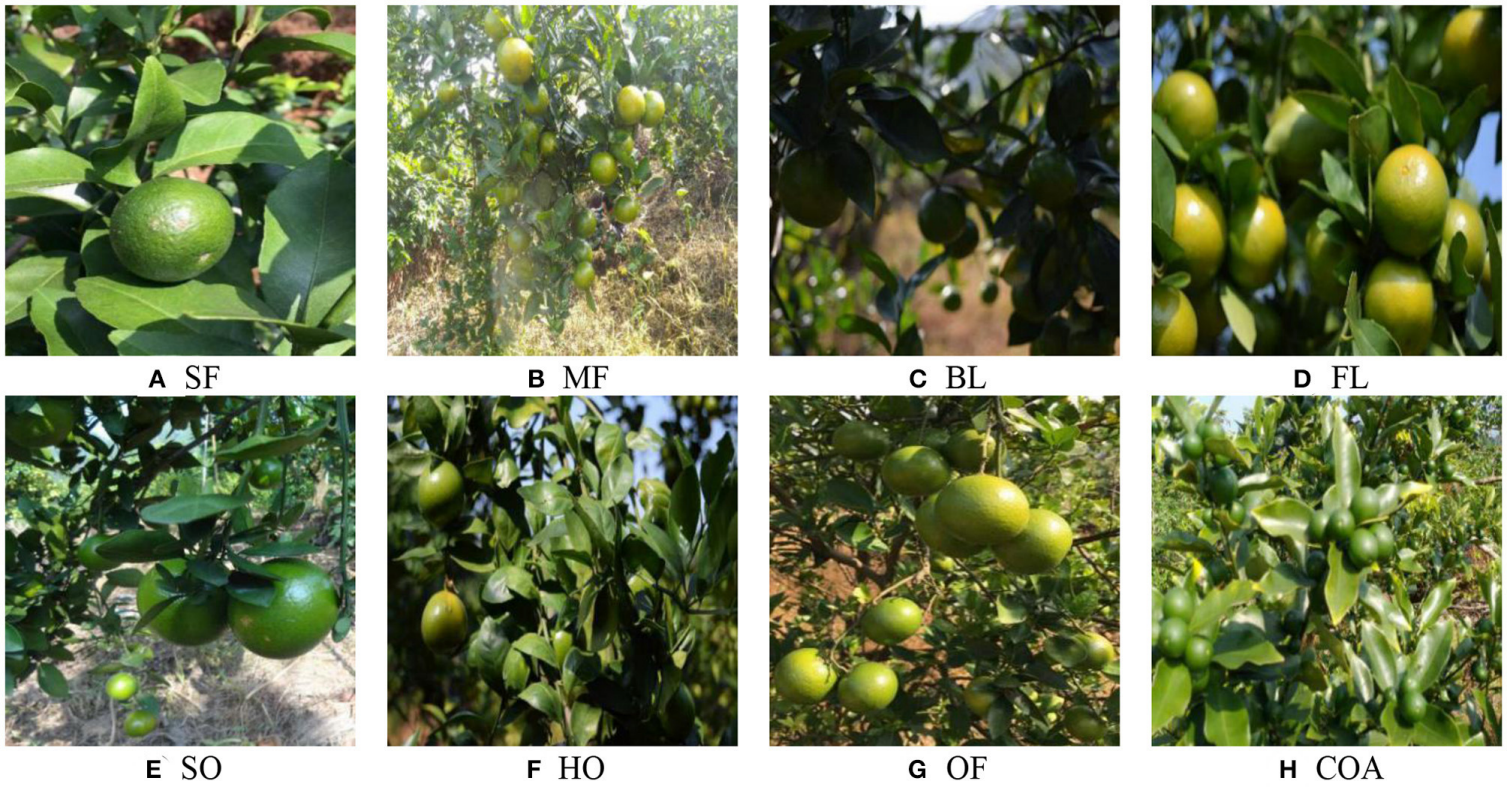
Classification and definition of different citrus scenes in natural environment.

To verify the average detection accuracy of green citrus fruits under the different conditions discussed using the algorithm, we defined and classified the citrus image set shown in Figure 2. The distribution of citrus fruits can be classified into single and multiple fruits, shown in Figures 2A,B. Figures 2C,D show different fruit illuminations, which can be divided into backlighting and front lighting. The cases of citrus fruits occluded by leaves or branches are shown as Figures 2E,F. In particular, if more than one-third of the fruit was occluded, it was called heavy occlusion (Figure 2F); otherwise, it was a slight occlusion (Figure 2E). Figure 2G shows the overlapping of multiple citrus fruits. In addition, citrus fruits are often presented in a combination of the mentioned conditions when they were in the actual orchard environment. Figure 2H contains four conditions, including multiple small-size fruits, backlighting, branch and leaf shading, and fruit overlapping. The classification and naming of the discussed cases are shown as Table 2.

**Table 2:** Green citrus scene classifications and image quantities.

**Class name**	**Green citrus scene**	**Images type**	**Number of images**
SF	Growth distribution	Single fruit	1,146
MF		Multiple fruits	3,298
FL	Illumination change	Front-lighting	2,767
BL		Back-lighting	1,776
SO	Occlusion by mixture of leaves and branches	Slight occlusion	3,045
HO		Heavy occlusion	1,320
OF	Overlap between fruits	Overlapping fruit	3,331
COA	Combination of the above	Combination of the above two or three cases	1,094

### Image Data Augmentation

To improve the generalization ability of the detection algorithm, this study adopted four methods to enhance the data set, including rotation, horizontal inversion, adding Gaussian noise, and changing brightness. Then, we removed invalid images without fruits or blurs and counted the number of images of each citrus scene. The statistical results are shown in Table 2. Finally, the dataset was randomly divided into training, validation, and test sets according to the proportions 70, 15, and 15%, respectively.

## Methodology

### Experimental Environment

The running environment included a desktop computer with Intel i7-10700 (2.9 GHz × 16) octa-core CPU, NVIDIA GeForce GTX 3090 GPU, and Ubuntu 16.04 64-bit systems. The software included CUDA 10.0.130, CUDNN 7.4.2, NVIDIA driver 410.78, Opencv 3.4.5, Deep Learning Framework-DarkNet, labelImg, spyder, and Anaconda 3.5.2.

### YOLO v4 Network

Compared with other object detection methods [such as regional-based convolutional neural network (R-CNN) series (Ren et al., 2015; He et al., 2017; Liang et al., 2020)], YOLO v4 (Bochkovskiy et al., 2020) extracts features based on a regression method, with a single neural network being used to detect and classify input images without generating a large number of candidate windows in order to realize end-to-end object detection. The YOLO v4 algorithm can quickly predict and classify targets and ensure high accuracy simultaneously, so it is suitable for application in actual environments.

**Figure 3:**
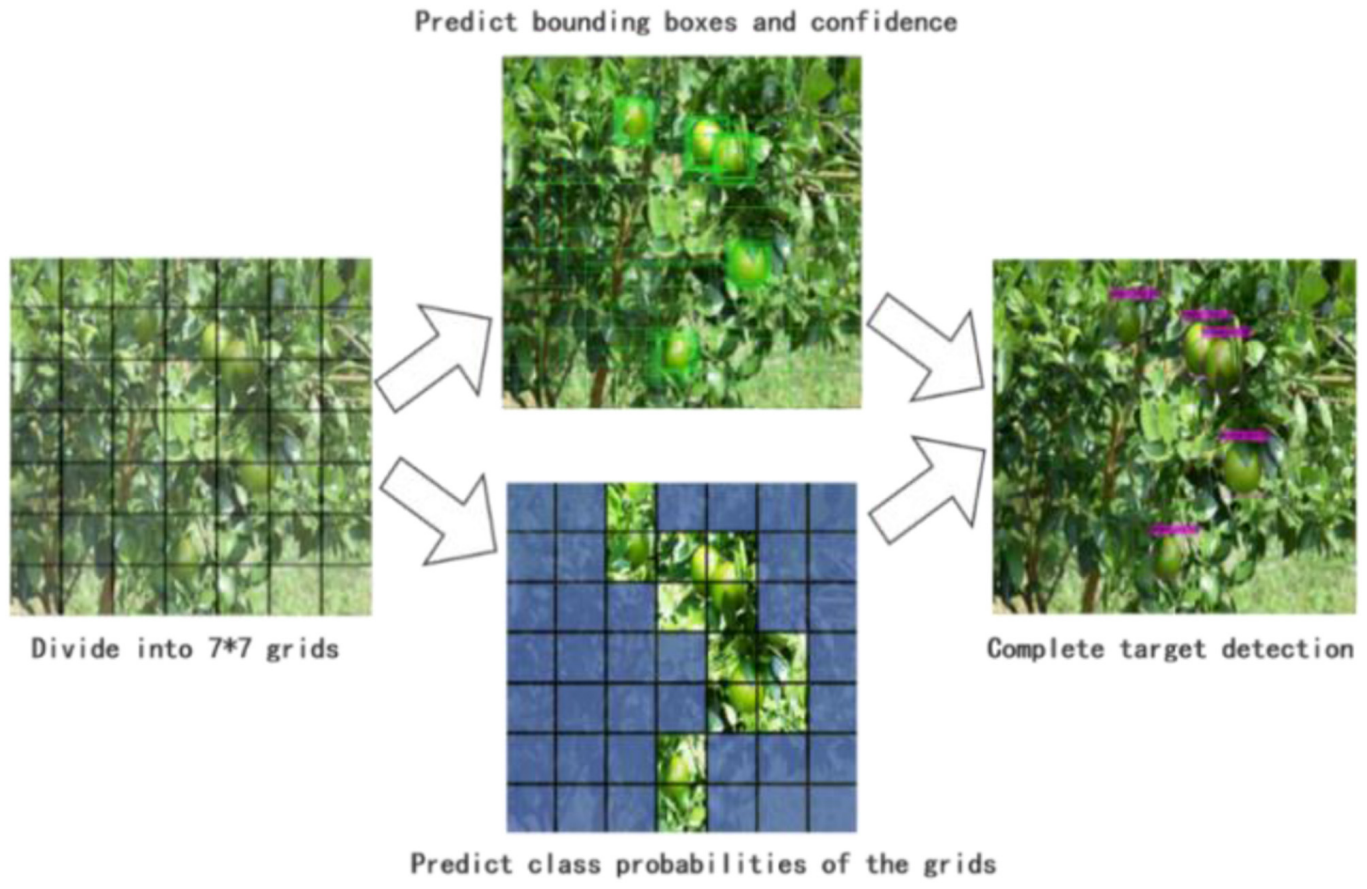
YOLO V4 detection network.

Shown as Figure 3, the YOLO v4 divides the input image into grids with a size of **S× S (S=7)**. If the center of an object falls within a certain cell, the cell is responsible for detecting the object. The cell outputs multiple prediction boxes and their corresponding confidences, discards the prediction boxes with low confidences, and, finally, locates the citrus position by the non-maximum suppression algorithm (Lawal, 2021).

### Improved YOLO v4 Network

The YOLO v4 is the fourth version of the YOLO algorithm series. Its average precision (AP) and FPS achieved 43.5 and 65%, respectively, on the Microsoft Common Objects in Context (MS COCO) dataset, resulting in a better performance than the Faster R-CNN (Ren et al., 2015), SSD (Liu et al., 2016), EfficientDet (Tan et al., 2020), and other object detection algorithms. However, we found that YOLO v4 had some shortcomings during the experiment, such as the missed detection of overlapped foreground objects and the incorrect detection of background objects. Although the backbone of YOLO v4 performed well, at the same time, its network was too deep, which would result in huge computation. Therefore, based on the improvements of YOLO V4, this study proposed a green citrus detection network named YOLO BP, which effectively improved the accuracy and real-time performance of the detection algorithm.

#### Feature Extraction Network

In view of the difficult and challenging nature of green citrus detection in natural environments, the accuracy of the visual system of picking robots should be improved as much as possible on the premise of real-time algorithm performance. Compared with the lightweight and high-speed YOLO v4_Tiny algorithm, the YOLO v4_CSPDarknet53 pursues better performance while ensuring real-time performance. Thus, referring to the Cross Stage Partial Network (CSPNet) (Wang et al., 2019), the CSPDarknet53 was proposed on the basis of the improved Darknet53. Furthermore, as the layer depth increases, the network performance will quickly reach saturation or even begin to decline, slowing the network reasoning speed and, at the same time, increasing the computation cost. Therefore, after many instances of clippings and experimental verifications, the CSPDarknet56 was finally proposed as the feature extraction network of YOLO BP.

**Figure 4:**
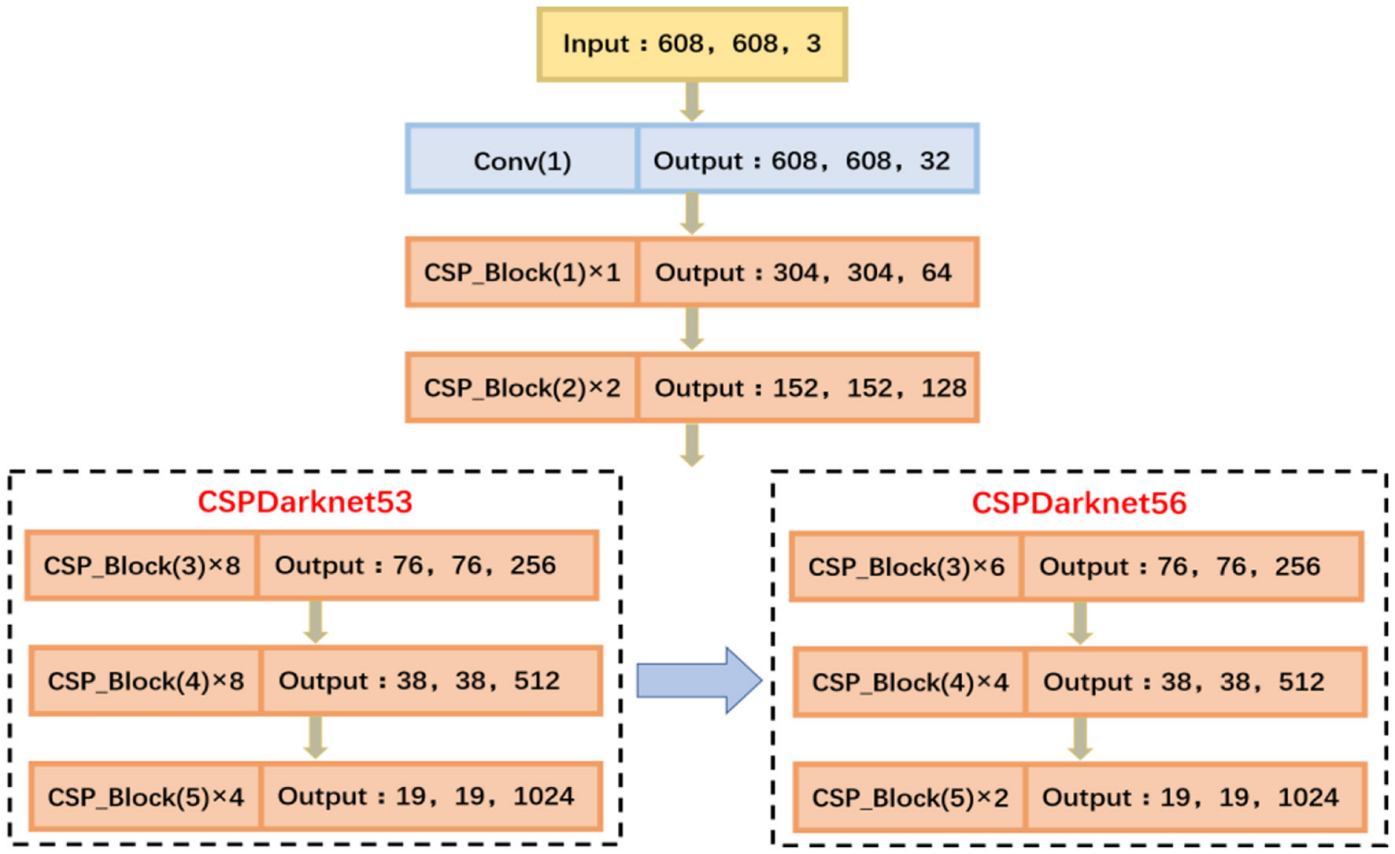
Feature extraction network.

Shown as Figure 4, the feature extraction network CSPDarknet56 contains five CSP_Blocks, each with a 3^*^3 convolution kernel used for downsampling. Compared with the CSPDarknet53, which contains 72 convolutional layers, the CSPDarknet56 proposed in this study has the following two advantages: (1) 16 convolutional layers have been clipped and the deep network can extract high-quality semantic information more efficiently, realizing the reuse and fusion of multilayer characteristics; (2) the memory cost is reduced and the network becomes lightweight on the premise of a high detection accuracy, resulting in the effective improvement of detection speed.

#### Feature Enhancement and Fusion Network

In order to further improve the performance of the detection network, researchers usually add some convolutional layers between the feature extraction network and the output layer to better integrate features. To increase the receptive field to a greater extent, Spatial Pyramid Pooling Network (SPP-Net) (He et al., 2015) was added after the CSPDarknet53 of YOLO v4. The added network can effectively separate most of the significant context features and hardly affect the computing speed.

**Figure 5:**
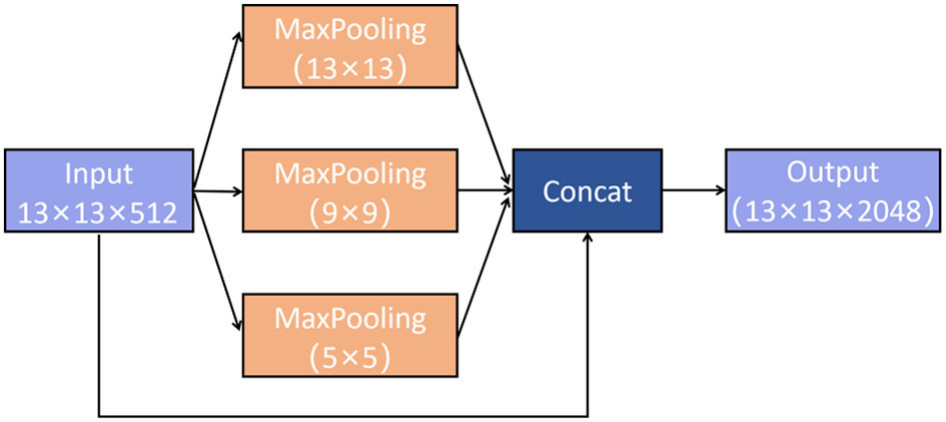
Spatial Pyramid Pooling Network (SPP-Net).

Shown as Figure 5, using convolution kernels with the sizes of {1*1, 5 * 5, 9 * 9, 13 * 13}, respectively, SPP-Net performs maximum pooling on the feature map obtained in the previous stage, and then aggregates all the feature maps through the concat layer.

**Figure 6:**
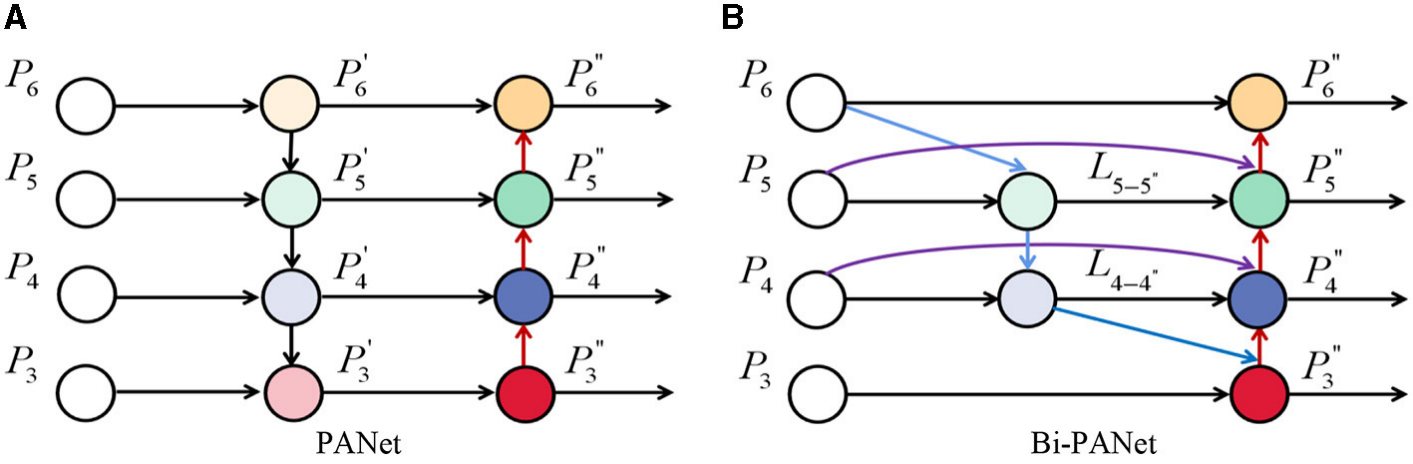
**(A,B)** Represent Path Aggregation Network (PANet) and bi-directional feature pyramid network (Bi-PANet).

In addition, PANet (Liu et al., 2018) was adopted as a parameter aggregation method in YOLO v4, shown as Figure 6A. Although PANet proves the effectiveness of two-way fusion, it ignores the possibility that the contributions of the different levels of features may be different. In order to reuse and fuse multilayer features more efficiently and get better detection performance, referring to the idea of a BiFPN network (Tan et al., 2020), this paper proposed a feature fusion network named Bi-PANet. The structure of Bi-PANet is shown as Figure 6B.

Compared with PANet, Bi-PANet was improved as follows: (1) to reduce unnecessary cost of computations, the PANet was simplified by removing redundant nodes from both ends of the network, as **P^′^_3_** and **P^′^_6_**, as shown in Figure 6A; (2) to strengthen the reuse and fusion of multilayer features, the extra edges from the original feature nodes to the output nodes were added as $L_{4-4^{\prime }}$ and $L_{5-5^{\prime }}$, as shown in Figure 6B.

#### YOLO BP Network

**Figure 7:**
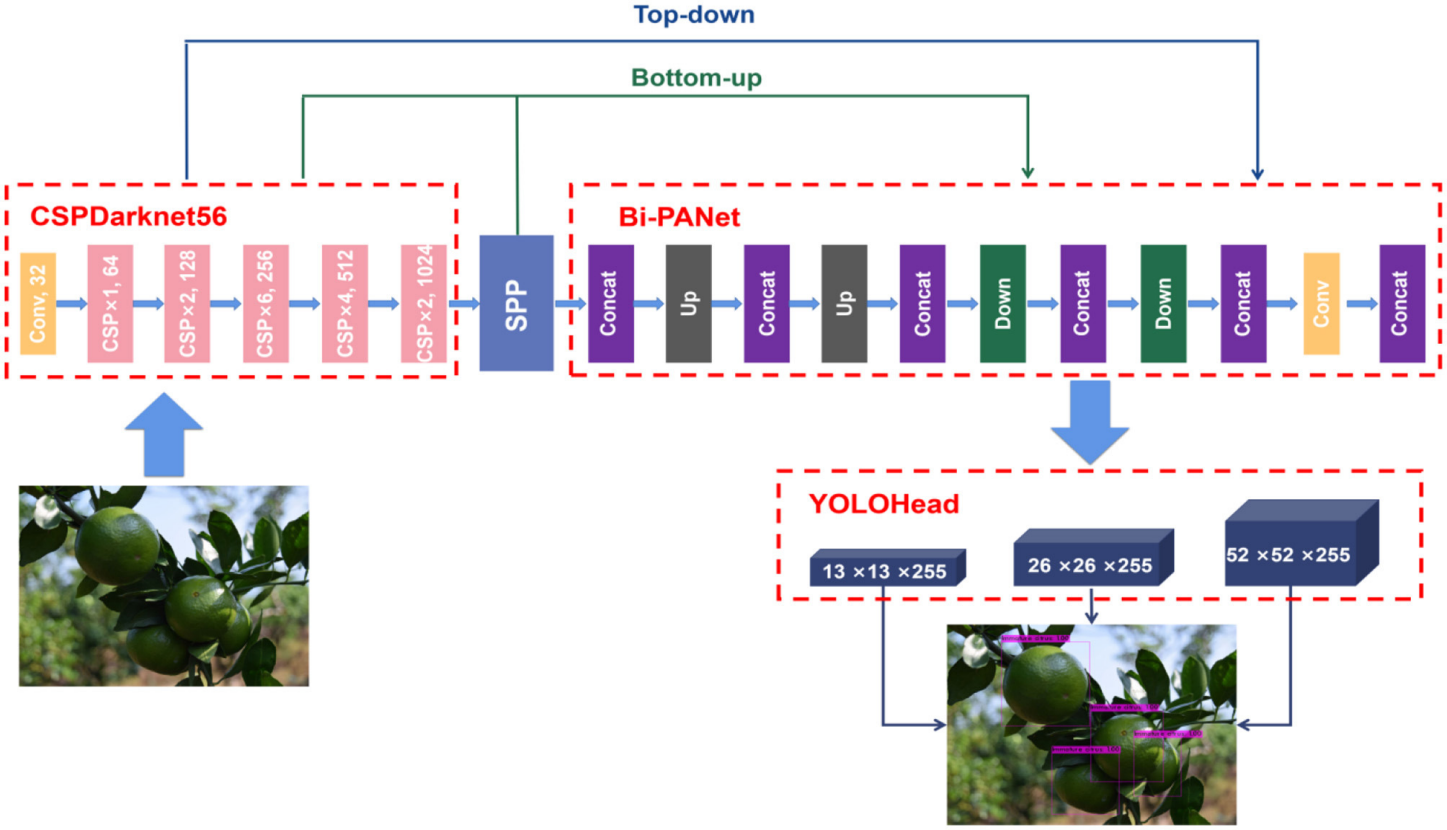
YOLO BP network architecture diagram.

Through the improvements of the backbone and feature fusion network, this study proposed a green citrus detection network based on YOLO BP, shown as Figure 7. Firstly, the CSPDarknet56 was used to extract the features of the input images in the backbone network. By reusing CSP_Blocks, the features of the shallow convolution layer could better and faster transmit to the deep convolution layer, resulting in the whole network having high information transmission and gradient transmission efficiencies. Secondly, to improve the ability of the network to extract deep features, the SPP-Net was used to perform maximum pooling and aggregation with different scales on the feature map, with the ability improvement of the deep layer feature selection. Then, the Bi-PANet was used to perform upsampling and downsampling operations twice for each technique. It also spliced the corresponding feature maps in the network, achieving the effective reuse and fusion of features. Finally, the regression prediction was performed three times in the prediction network to realize the multi-scale detection of green citrus fruits with different sizes.

## Experimental Results and Analysis

### Model Training

The training setting and strategy are as follows:

(1) The hyper-parameters setting. The training steps were 20,000, the batch size was 64, and the momentum factor and weight decay were 0.949 and 0.0005, respectively. The step decay learning rate scheduling strategy was adopted with an initial learning rate of 0.001 and the learning rates of 0.0001 and 0.00001 at the steps 16,000 and 18,000, respectively.
(2) Training strategy. The weight parameters of the pre-trained network (yolov4.conv.137) were used for initialization. The multi-scale training strategy and mosaic data augmentation method were adopted to improve the robustness and accuracy of the network for images with different resolutions.

**Figure 8:**
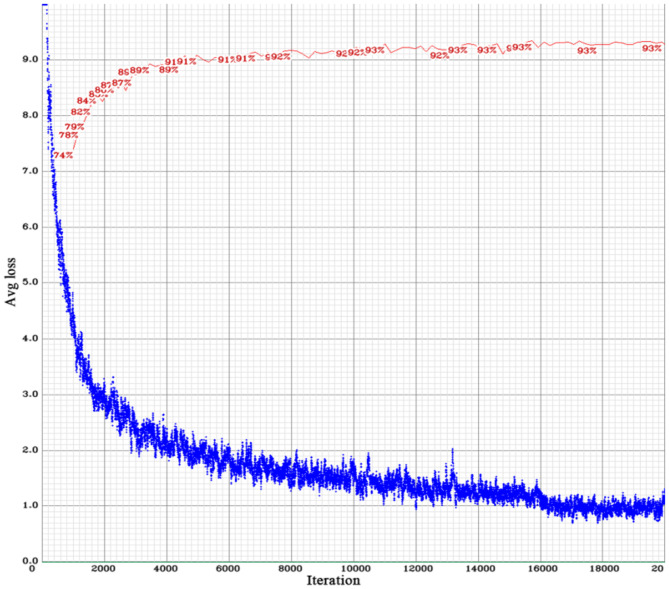
Curves of the loss function value and mean average precision (mAP) of YOLO BP network.

**Figure 9:**
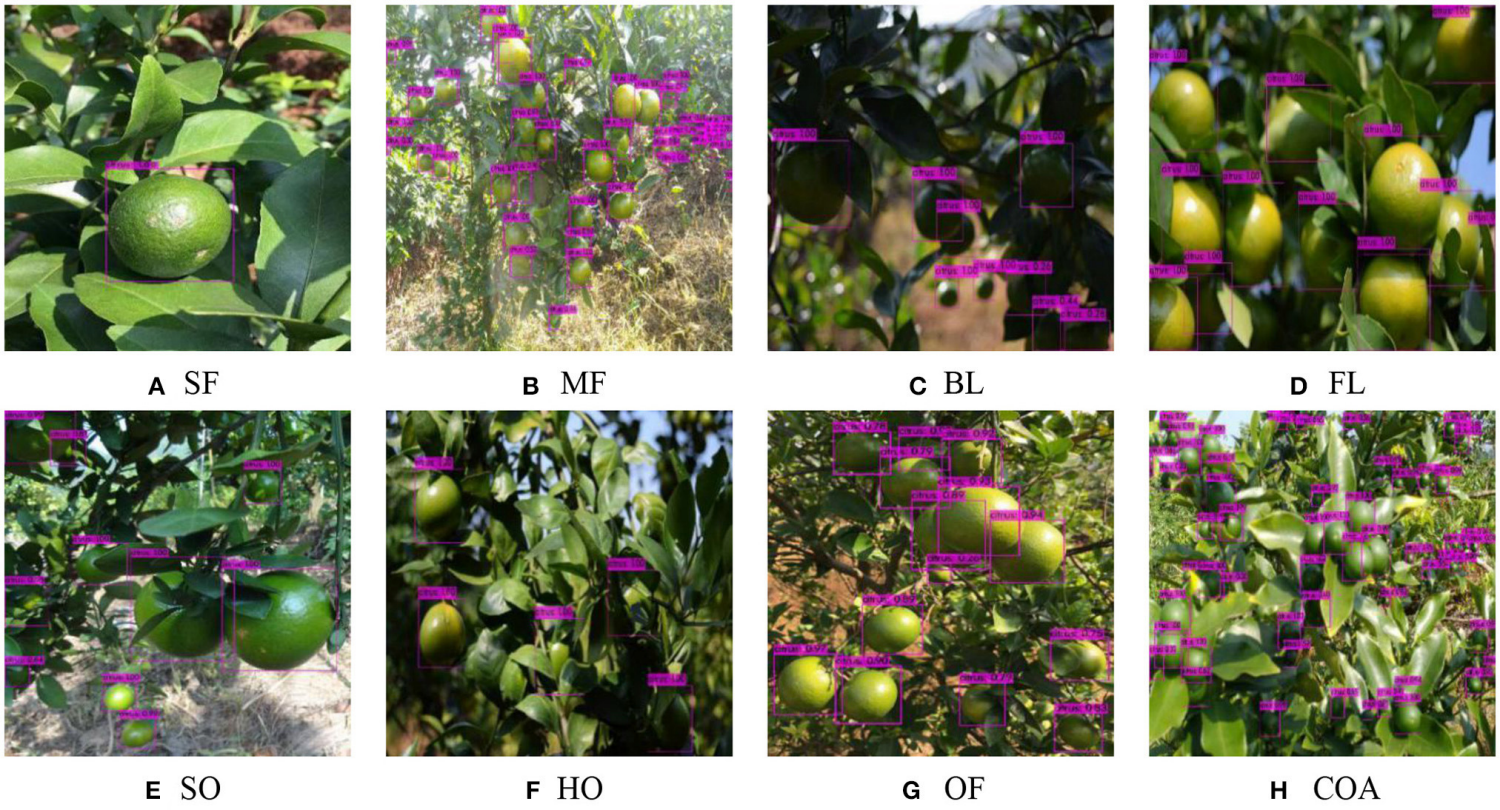
Green citrus fruit detection results under different conditions.

Figure 8 shows the change curves of the average loss and mAP values of the YOLO BP network during training. The network updated the loss function of the small sample batch, resulting in a gradual decrease of prediction loss deviation. When the network training steps exceeded 16,000, the loss value and mAP were basically stabilized with approximately 1 and 93%, respectively. From the point of parameter convergence, the result of the network training was satisfactory. Figure 9 shows the results of the detection of citrus fruits in various environments by the trained YOLO BP network.

### Experiment and Evaluation of Citrus Detection

To verify the effectiveness of the proposed detection algorithm, three groups of performance comparison experiments for the green citrus recognition algorithm were designed in this study.

The precision (*P*), recall (*R*), and F1 score (*F*_1_) are defined as follows:

(1) $$\begin{array}{rcl} P & = & \frac{T_{P}}{T_{P}+F_{P}} \end{array}$$

(2) $$\begin{array}{rcl} R & = & \frac{T_{P}}{T_{P}+F_{N}} \end{array}$$

(3) $$\begin{array}{rcl} F_{1} & = & \frac{2PR}{P+R} \end{array}$$

Where *T*_*P*_, *F*_*P*_, and *F*_*N*_ are true positive, false positive, and false negative, respectively.

#### Comparative Experiment of YOLO BP and YOLO v4

The performances of the detection algorithms were uneven in different situations. As shown in Figure 2A, the fruits are relatively clear, complete and easy to identify. However, with backlighting, the detection of small-size fruits, overlapping fruits, or fruits covered by branches and leaves would be more difficult, which is shown in Figure 2G. Therefore, the experiment designed in this section mainly focused on the detection performance of YOLO BP and YOLO v4 in complex scenarios. The test set included multiple fruits (MF), backlighting (BL), slight occlusion (SO), heavy occlusion (HO), overlapping fruits (OF), and the combination of the above (COA). The hyper-parameters setting and training strategy of the YOLO v4 network were consistent with those of YOLO BP network.

**Figure 10:**
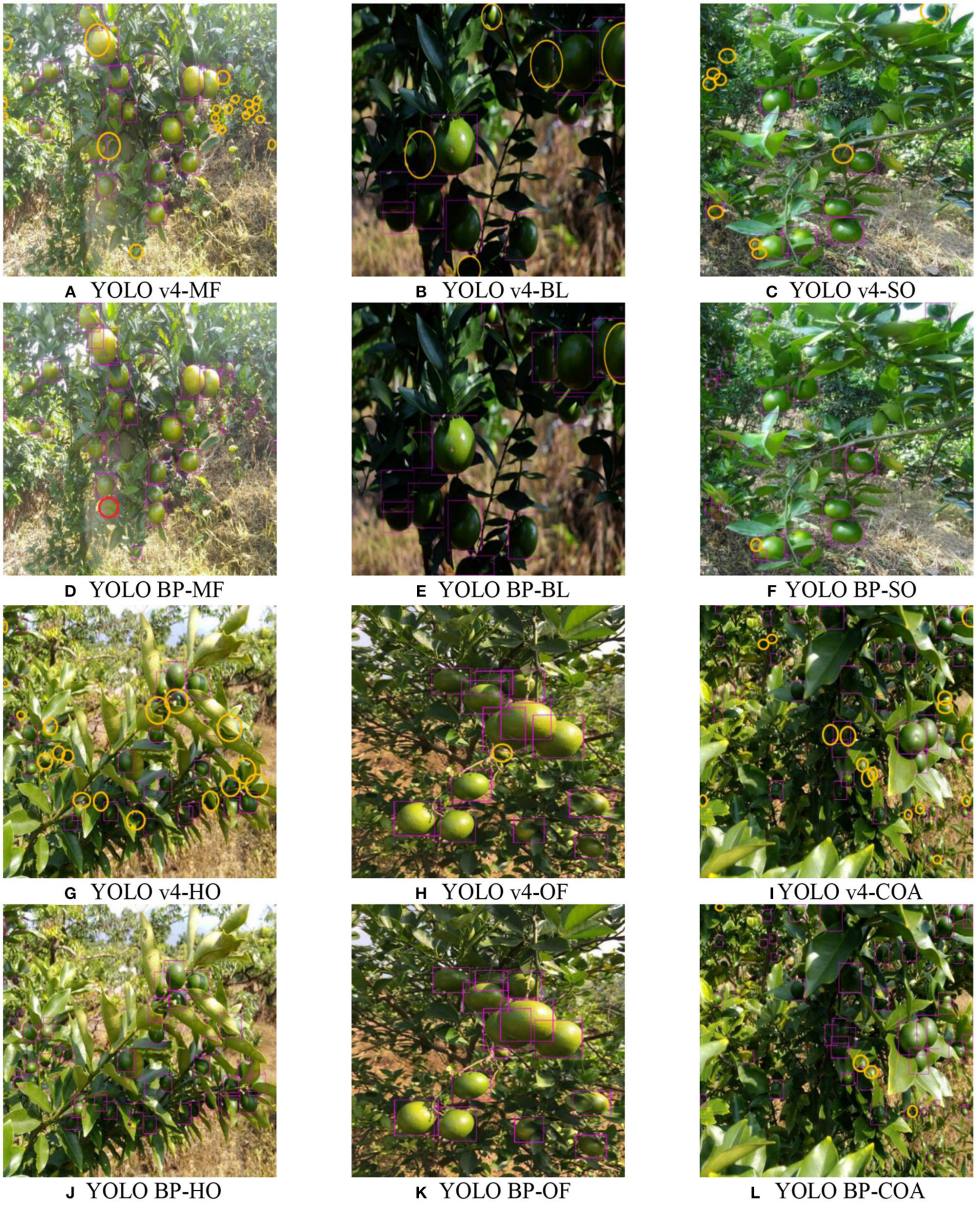
The comparison between the proposed method and the traditional YOLO v4. **(A–C)** and **(G–I)** are the detection results of the traditional YOLO v4. **(D–F)** and **(J–L)** are the detection results of YOLO BP.

Figure 10 shows the detection results of the two algorithms. The missed and false detections are manually marked with yellow and red ellipses, respectively.

As shown in Figure 10, both YOLO BP and YOLO v4 can detect complete, slightly occluded, or overlapping green citrus fruits. However, YOLO BP (e.g., Figures 10D,F,L) can overcome the defect of missing small objects that is present in YOLO v4 (e.g., Figures 10A,C,I). Compared with YOLO v4 (such as Figures 10B,I), YOLO BP (such as Figures 10E,L) can also better detect citrus fruits that overlapped or were occluded by leaves under the backlighting environment. In addition, compared with YOLO v4 (such as Figures 10G,I), YOLO BP (such as Figures 10J,L) can detect citrus fruits heavily occluded by branches and leaves with better robustness and accuracy. The experimental results showed that the overall performance of YOLO BP was better than YOLO v4.

**Figure 11:**
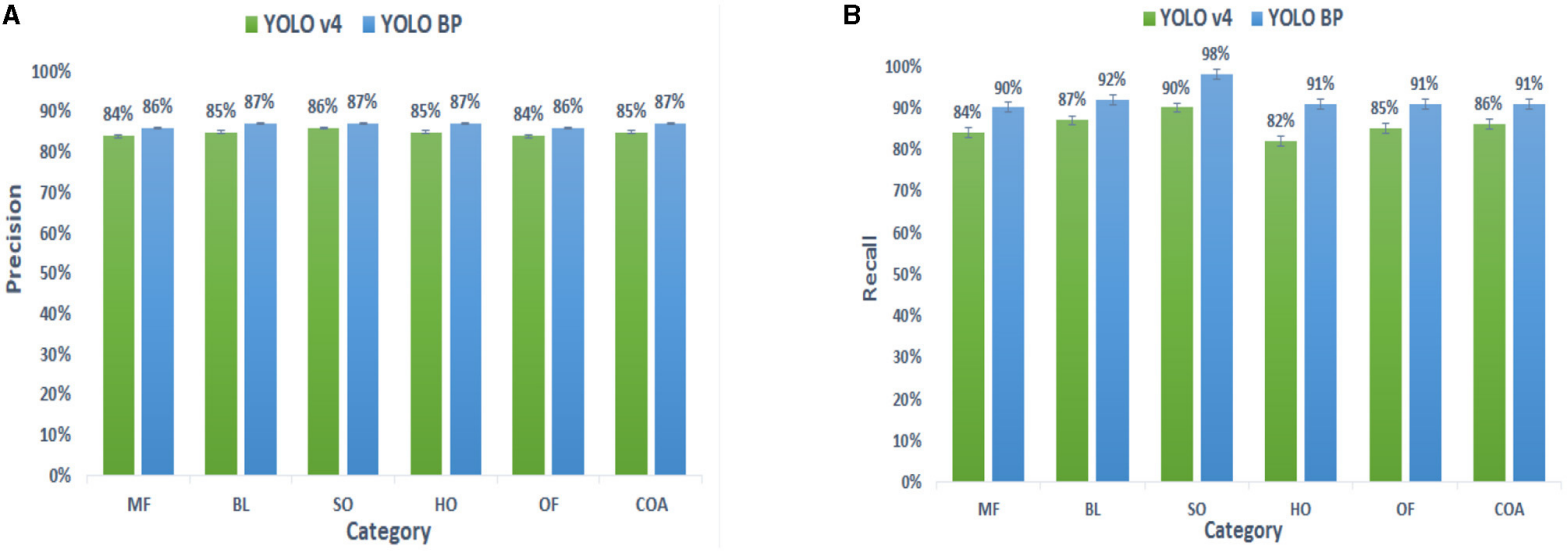
**(A,B)** Represents the comparison of precision and recall of YOLO BP and YOLO v4.

Figure 11 and Table 3 show the comparison results of YOLO BP and YOLO v4 in six different complex citrus scenes. It can be seen that the accuracy, recall, mAP, F1 score, and average IoU of the YOLO BP recognition algorithm are higher than those of YOLO v4. Especially in the HO case, the mAP of YOLO BP was nearly 6% higher than that of YOLO v4. The experimental results showed that, in the six gradient test sets conducted, the average mAP, average IoU, and average F1 score of the YOLO BP reached 92.91 and 75.47% and 0.89, respectively, which were higher than the 88.57 and 69.5% and 0.85 of YOLO v4, indicating that YOLO BP has better detection effect.

**Table 3:** YOLO v4 and YOLO BP performance comparison under different citrus situations.

**Citrus situations**	**mAP (%)**		**F1**		**Average IoU (%)**	
	**YOLO v4**	**YOLO BP**	**YOLO v4**	**YOLO BP**	**YOLO v4**	**YOLO BP**
MF	86.31	90.80	0.84	0.88	68.41	74.37
BL	89.03	92.50	0.86	0.89	71.14	78.15
SO	93.43	97.44	0.88	0.92	71.40	77.57
HO	86.59	92.54	0.84	0.88	68.23	73.04
OF	87.24	91.46	0.84	0.88	69.29	76.65
COA	88.82	92.71	0.85	0.88	68.55	73.04
Mean	88.57	92.91	0.85	0.89	69.50	75.47

#### Precision and Real-Time Analyses of the Citrus Detection Network

**Figure 12:**
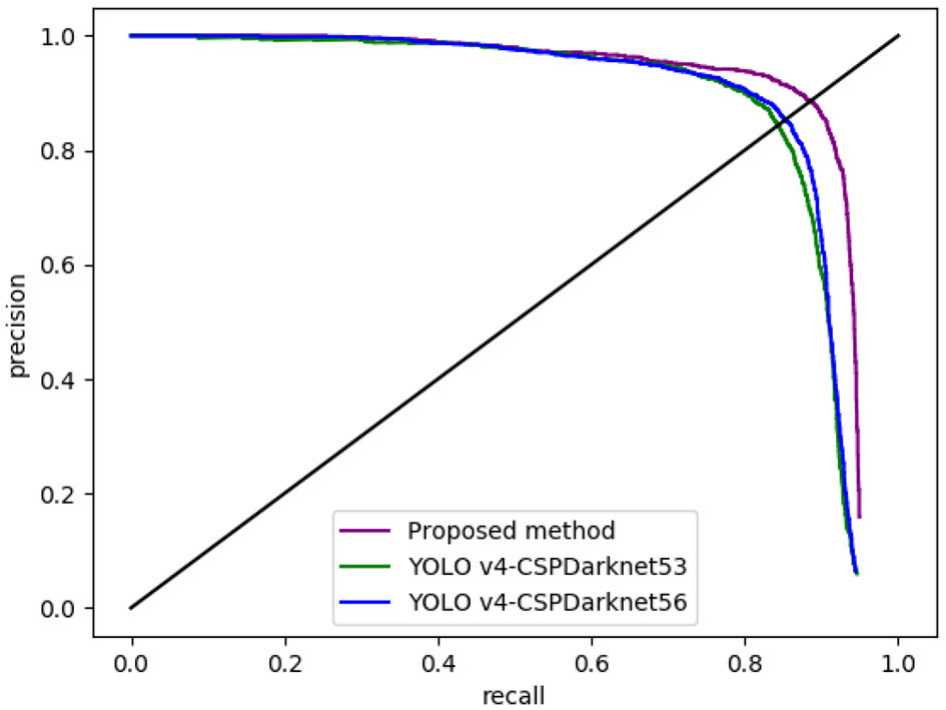
The comparison of precision and recall curves of the three algorithms.

To further evaluate the contributions of CSPDarknet56 and Bi-PANet, a group of comparative experiments of YOLO v4–CSPdarknet53, YOLO v4–CSPdarknet56, and YOLO BP were designed. The above three algorithms were used to detect the whole original test sets. The accuracy, recall, mAP, and FPS of the three algorithms were computed, shown as Table 4, and the precision and recall curves of the three algorithms were obtained, shown as Figure 12.

**Table 4:** Disentangling backbone and Bi-PANet—starting from the standard CSPDarknet (CSPDarknet53 + PANet), we first replace the backbone with CSPDarknet56 and then replace the baseline PANet with our proposed Bi-PANet.

**Detection methods**	**Precision**	**Recall**	**mAP**	**FPS**
YOLO v4-CSPDarknet53(CSPDarknet53+SPP+PANet+YOLO Head)	84%	84%	87.25%	17
YOLO v4-CSPDarknet56(CSPDarknet56+SPP+PANet+YOLO Head)	84%	86%	8816%	19
The proposed method(CSPDarknet56+SPP+Bi-PANet+YOLO Head)	86%	91%	91.55	18

Figure 12 shows that the precision-recall (PR) curve of the YOLO v4-CSPDarknet53 is under the PR curves of YOLO v4–CSPDarknet56 and YOLO BP, indicating that the performance of the latter two algorithms is better than YOLO v4–CSPDarknet53.

It can be seen from Table 4 that, compared with the original YOLO v4-CSPDarknet53, the mAP of YOLO v4-CSPDarknet56 is 88.16% and FPS effectively increased to 19 frames per second, showing that the improved feature extraction network contributed 0.91 percentage points of mAP and 2 frames of FPS. Compared with YOLO v4–CSPDarknet56, the mAP and FPS of the proposed method reached 91.55% and 18 frames, indicating that the improved feature-enhanced fusion network contributed 3.39% of mAP. The final results showed that, compared with YOLO v4, the mAP and FPS of YOLO BP were increased by 4.3% and 1 frame, respectively, indicating the higher detection speed and accuracy of the proposed method.

#### Comparison Experiment of Different Green Citrus Detection Methods

To further verify the performance of the proposed method, we also designed a comparative experiment of existing green citrus detection algorithms, namely, the DBBRF (He et al., 2020), Faster RCNN (Xiong et al., 2018), and the proposed algorithm. The average accuracy, F1 value, and execution time were the evaluation indicators, as shown in Table 5.

**Table 5:** Comparison of green citrus detection algorithms.

**Detection algorithms**	**mAP (%)**	**F1 (%)**	**Time/s**
Deep Bounding Box Regression Forest (He et al., 2020)	87.60	85.38	0.759
VGG16+Faster R-CNN (Xiong et al., 2018)	85.49	85.31	0.400
The proposed method	91.55	88.00	0.057

Firstly, by comparing the two existing methods in Table 5, we found that mAP and F1 of the DBBRF proposed by He et al. were 87.6 and 85.38%, respectively, which were higher than the 85.49 and 82.31% of Faster R-CNN, which was proposed by Xiong et al. However, the detection speed of the former was 0.759 s per frame, which was lower than the 0.4 s of the latter. Compared with the DBBRF and Faster R-CNN, the mAP, F1, and execution time of the proposed method were 91.55 and 88% and 0.057 s, respectively, showing a greater advantage in performance. The experimental results showed that the proposed detection algorithm for green citrus has strong robustness and high accuracy in the complex orchard environment, which provides technical support for green fruit detection in natural environments.

## Discussion

Compared with the original algorithm YOLO v4 (one-stage detection network), it can be seen from Figure 11 and Table 3 that the accuracy, recall, map, F1 score, and average IOU of YOLO BP are higher than YOLO v4. The main reasons are as follows: (1) the backbone network (CSPDarknet56) was proposed, which could extract higher quality features; (2) a bi-directional feature pyramid network (Bi-PANet) was proposed by removing the redundant nodes of PANet and adding additional connections, which could enhance the robustness of small-size and overlapping fruit detection effectively. Moreover, from the PR curve (Figure 12) and Table 4, we can observe that CSPDarknet56 and Bi-PANet proposed in this study both contribute to the improvement of network detection performance, indicating the feasibility and effectiveness of our method.

Compared with the DBBRF proposed by He et al. (2020) and Faster R-CNN (two-stage detection network) proposed by Xiong et al. (2018), it can be seen from Table 5 that YOLO BP has high accuracy and real-time performance in complex environments. Analyzing the experimental results, we found that the good performance of YOLO BP benefitted from the following aspects: (1) the strong feature extraction network and feature enhancement and the fusion network; (2) the real-time advantage of the deep learning algorithm (especially the one-stage method). The experimental results showed that the proposed method had a greater advantage in performance, meeting the accuracy and real-time requirements of picking robots.

Although the proposed method had strong robustness and high accuracy in the complex orchard environment, there were also some shortcomings, as shown in Figure 10. By analyzing the results of the comparative experiments, the main possible reasons affecting the detection accuracy were concluded as follows; (1) leaves with similar color and shape to citrus were easily mis-detected as citrus, shown as the red ellipse mark in Figure 10D; (2) in the backlight environment, multiple fruits with severe overlap were easily identified as one fruit, shown as the yellow ellipse mark in Figure 10E; (3) some citrus fruits in the image were too small and occluded by branches and leaves at the same time, which led to missed detections, shown as the yellow ellipse marks in Figure 10L.

## Conclusions and Future Work

In this study, we proposed a multi-scale convolutional neural network named YOLO BP to detect green citrus fruits in natural environments. First, we carried out image acquisition experiments of green citrus fruits and classified data sets. Second, four methods were implemented to enhance the citrus image set. Moreover, we improved the structure of the YOLO V4 framework and made progress on the detection of green citrus in complex scenarios. Finally, by conducting three groups of comparison experiments, the proposed method was proven to achieve better recall and accuracy.

In conclusion, the proposed method provided a new visual recognition idea for fruit-picking robots and fruit-picking unmanned aerial vehicles (UAVs). Although this research made some progress, there are still some issues to be improved upon, such as the deficiency of the proposed method mentioned at the end of discussion, which is the necessary manual labeling and lack of samples. For further research, we will focus on solving these problems in order to better apply deep learning methods to the visual detection methods of picking robots.

## Data Availability Statement

The raw data supporting the conclusions of this article will be made available by the authors, without undue reservation.

## Author Contributions

All authors contributed to the conception and design of the study, dataset generation, model training and testing, analysis of results, and the drafting, revising, and approving of the contents of the manuscript.

## Funding

This research was funded by National Natural Science Foundation of China (No. 32071912), Science and Technology Program of Guangzhou (202002030423), Guangzhou Key Laboratory of Intelligent Agriculture (201902010081), the High-level talents scientific research start-up fund project of Guangdong Mechanical and Electrical Polytechnic (Gccrcxm-201904), and the Special Funds for the Cultivation of Guangdong College Students' Scientific and Technological Innovation (Climbing Program Special Funds) (pdjh2021a0070).

## Conflict of Interest

The authors declare that the research was conducted in the absence of any commercial or financial relationships that could be construed as a potential conflict of interest.

## Publisher's Note

All claims expressed in this article are solely those of the authors and do not necessarily represent those of their affiliated organizations, or those of the publisher, the editors and the reviewers. Any product that may be evaluated in this article, or claim that may be made by its manufacturer, is not guaranteed or endorsed by the publisher.
